# Reply to Condello I

**DOI:** 10.1093/icvts/ivae150

**Published:** 2024-08-29

**Authors:** Tiago R Velho, Rafael M Pereira, Luís F Moita

**Affiliations:** Cardiothoracic Surgery Department, Hospital de Santa Maria, Unidade Local de Saúde de Santa Maria, Lisbon, Portugal; Cardiothoracic Surgery Research Unit, Faculdade de Medicina da Universidade de Lisboa, Centro Cardiovascular da Universidade de Lisboa (CCUL@RISE), Lisbon, Portugal; Cardiothoracic Surgery Research Unit, Faculdade de Medicina da Universidade de Lisboa, Centro Cardiovascular da Universidade de Lisboa (CCUL@RISE), Lisbon, Portugal; Escola Superior de Saúde da Cruz Vermelha Portuguesa, Lisbon, Portugal; Center for Disease Mechanisms Research, Faculdade de Medicina da Universidade de Lisboa, Lisbon, Portugal

**Keywords:** Cardiopulmonary bypass, Postoperative organ dysfunction, Sequential Organ Failure Assessment Score, Cardiac surgery

We read with interest Condello’s [1] comments on our recent publication. We agree with Condello that cardiopulmonary bypass (CPB) duration alone is not the sole determinant of patient outcomes. However, the primary goal of our study was to determine the impact of CPB time on the Sequential Organ Failure Assessment (SOFA) score after cardiac surgery [2].

CPB induces a significant immunologic and inflammatory response that affects the function of virtually all tissues and organs. In our study, only 17.2% of patients showed no organ dysfunction, as assessed by the SOFA score. Beside the immediate impact of postoperative organ in the Intensive Care Unit, we have previously demonstrated that the SOFA score at the Intensive Care Unit also predicts mortality 12 and 24 months after surgery [3].

Undeniably, advances in cardiac surgery rely on innovations in CPB to achieve better outcomes and reduce associated morbidity. Therefore, research is warranted in multiple fields, including venous return techniques, optimized cannulation strategies, intraoperative management of oxygen delivery and haemoglobin, and biocompatibility, among others. CPB improvements, combined with new surgical techniques and potential pharmacological interventions, may provide better clinical benefits [4].

To develop ground-breaking interventions, we need tools to evaluate and stratify morbidity accurately. Additionally, strong evidence shows that postoperative morbidity and mortality correlate directly with CPB duration. Considering these factors, we used CPB duration as a variable to understand how it influences the overall SOFA score and for each of its systems individually.

Our article highlights the significance of the SOFA score as a valuable tool for directly assessing and classifying CPB-related postoperative organ dysfunction. Moreover, our model also has the advantage of providing the predicted probabilities for the impact of the overall SOFA score and for each of the 6 systems, according to CPB time. This enables using the SOFA score to measure organ dysfunction after CPB. New approaches and comparative studies can use the SOFA score to evaluate the impact of interventions. Our study’s major contribution is providing a quantitative tool to score organ dysfunction after CPB.

The statistical models used in our original research were specifically designed to address the primary objective of the study. Thus, the probability estimates for each SOFA category should not be generalized to other contexts. These estimates were specifically calculated to assess the increasing impact of CPB time on the dysfunction levels within each of the SOFA score systems in the analysed sample.

In conclusion, beyond measuring the impact of CPB duration on the SOFA score and its contribution to postoperative organ dysfunction, our main goal was to provide a valuable and easy tool for measuring the impact of CPB on organ dysfunction. This opens the door to using it as a quantitative outcome in future research.
